# HRV-Based Recognition of Complex Emotions: Feature Identification and Emotion-Specific Indicator Selection

**DOI:** 10.3390/healthcare13233036

**Published:** 2025-11-24

**Authors:** Da-Yeon Kang, Chan-Il Kim, Jong-Ha Lee

**Affiliations:** 1 Department of Biomedical Engineering, School of Engineering, Keimyung University, Daegu 42601, Republic of Korea; k.dayeon@inceptionlab.com; 2 InceptionLab Inc., Daegu 42601, Republic of Korea; ch.1@inceptionlab.com

**Keywords:** complex emotion, emotion recognition, HRV, HCI, mental health, healthcare, vision-based intelligence, rPPG, affective computing

## Abstract

**Background/Objectives:** Complex emotions in daily life often arise as mixtures of basic emotions, but most emotion-recognition systems still target a small set of discrete states and rely on contact-based sensing. This study aimed (1) to examine whether four compound emotions—Positive Surprise, Negative Surprise, Positive Sadness, and Negative Sadness—defined by valence direction within basic emotion categories can be differentiated using heart rate variability (HRV), and (2) to evaluate the feasibility of a camera-based contactless system (Deep Health Vision System, DHVS) by comparing it with a reference chest-strap device (Polar H10). **Methods:** Ten healthy adults viewed video clips designed to induce the four complex emotions. HRV was recorded simultaneously using Polar H10 and a webcam-based rPPG implementation of DHVS. Two-minute baseline and during-stimulus segments were extracted, and change rates of standard HRV indices were computed. After each stimulus, participants reported Valence, Arousal, Dominance, and proportional basic-emotion composition. Statistical analyses examined within-condition HRV changes, associations between HRV and self-reports, differences across emotion/valence conditions, and concordance between DHVS and Polar H10. **Results:** Self-reports confirmed distinct affective profiles for the four compound emotions. Positive and Negative Surprise were associated with heart rate reduction, while Positive Sadness showed reduced total power; Negative Sadness yielded heterogeneous but nonsignificant HRV changes. Specific HRV indices demonstrated condition-dependent correlations with Valence, Arousal, and Dominance. LF/HF changes were more sensitive to emotion category (Surprise vs. Sadness), whereas total power changes were more sensitive to valence (positive vs. negative). DHVS partially reproduced Polar H10 HRV patterns, with clearer concordance under positive-valence conditions. **Conclusions:** HRV captures distinct autonomic signatures of complex emotions defined by valence direction and shows meaningful links with subjective affective evaluations. LF/HF and total power provide complementary information on emotion category and valence-related autonomic reactivity, supporting indicator-specific modeling strategies. DHVS shows preliminary feasibility as a contactless HRV sensing platform for complex emotion recognition, warranting further validation with larger samples and more robust rPPG processing.

## 1. Introduction

With the growing demand for technologies capable of objectively and quantitatively recognizing human emotional states, artificial intelligence based emotion recognition systems have become increasingly important in fields such as human–computer interaction (HCI), mental health diagnosis and monitoring, and smart interface design. In particular, addressing the lack of mutual empathy in HCI remains one of the most critical challenges today. In an era of exponentially advancing technology, interfaces that fail to accurately detect a user’s emotional state and respond appropriately to contextual changes cannot be considered trustworthy. Therefore, to achieve genuine empathy in interactions, it is essential to establish methods that can interpret and understand users’ true emotions without requiring explicit input of their interpreted intent [1].

Recent studies indicate that most emotion recognition systems have been developed to classify basic emotions. However, findings from psychology and behavioral science have demonstrated that human emotions often emerge in more complex forms, with two or more emotions expressed sequentially or simultaneously. Individuals may experience complex emotions resulting from combinations of basic emotions [2,3]. For instance, sadness and joy may coexist, or conflicting emotions such as guilt and relief may be experienced simultaneously in daily life. Current emotion recognition models, which largely focus on predicting a limited set of discrete emotions, are therefore insufficient to capture the richness and fluidity of actual human affective states. This limitation reduces the ability of human–interface systems to precisely identify users’ emotional conditions and to respond in a timely and contextually appropriate manner. Moreover, emotion assessment methods based on facial expressions or verbal reports are constrained by intentional control, linguistic barriers, and expression difficulties among specific clinical populations—such as individuals with dementia, depression, schizophrenia, or autism spectrum disorder [4]. Consequently, emotion recognition approaches grounded in physiological signals—which cannot be easily controlled or consciously manipulated—offer a more reliable and objective alternative.

In this context, the present study aimed to overcome the limitations of existing emotion recognition systems that focus exclusively on basic emotion categories and fail to reflect the multidimensional nature of human affect. We selected sadness and surprise as representative emotions that can manifest in both positive and negative valence directions, defining four compound emotional conditions: Positive Surprise, Negative Surprise, Positive Sadness, and Negative Sadness. Physiological responses were analyzed using heart rate variability (HRV), which reflects the involuntary dynamics of the autonomic nervous system. Furthermore, the study examined the quantitative concordance between the Deep Health Vision System (DHVS)—a camera-based contactless biosignal acquisition system—and a reference contact-based device (Polar H10), to evaluate the feasibility of applying DHVS within emotion recognition frameworks. By moving beyond single-emotion classification and establishing a methodological foundation for HRV-based analysis of complex emotions, this study seeks to contribute to the development of more precise and high-dimensional emotion recognition systems, and to propose a scalable, contactless design architecture that expands the applicability of emotion recognition technologies in real-world environments.

## 2. Related Work

Recent studies on emotion recognition have proposed deep learning architectures that simultaneously learn spatiotemporal representations of EEG signals. For instance, STRFLNet integrates spatial patterns across EEG channels with temporal dynamics, thereby improving emotion classification performance [5]. Similarly, Fan et al. introduced a lightweight framework that combines residual convolution with capsule networks to efficiently extract local EEG features while preserving emotion-related representations [6]. These studies highlight that the key factor for improving performance lies in how emotional patterns in physiological signals are represented over time.

Although the present study employed HRV/rPPG-based contactless physiological signals rather than EEG, which offer lower signal resolution and information richness, the observed differences in HRV sensitivity across emotion categories and valence directions suggest that autonomic responses can also be structurally modeled over time. In future research, instead of using only condition-averaged HRV values as in this study, spatiotemporal representation learning or lightweight network architectures—similar to STRFLNet and capsule-based EEG approaches—could be applied to segment-level HRV time series to further enhance emotion recognition performance in contactless environments. In this respect, these two EEG-based studies, despite using different physiological modalities, share a common direction of “representation learning that preserves temporal dynamics,” providing a potential technological pathway to extend the findings of the present work.

## 3. Materials and Methods

### 3.1. Types of Complex Emotions

In this study, four types of complex emotions were defined according to the direction of valence: Positive Surprise, Negative Surprise, Positive Sadness, and Negative Sadness. Both surprise and sadness are emotions that, depending on context, can combine with opposing emotions to elicit complex affective responses. For example, an unexpected event in a favorable situation may induce Positive Surprise, whereas a startling event in a fearful context may result in Negative Surprise. Similarly, sadness evoked in an inspiring or touching situation may be classified as Positive Sadness, whereas sadness resulting from feelings of injustice or regret may be categorized as Negative Sadness. Thus, even when the same basic emotion is involved, the specific scenarios and causes can define a distinct complex emotion. These nuanced states reflect the authenticity of affective responses that conventional emotion recognition systems often fail to capture.

### 3.2. Stimuli for Inducing Complex Emotions

To effectively elicit the complex emotions defined in this study, we developed video clips based on validated emotional stimuli and established protocols for emotion induction used in previous research (Table 1) [7,8]. Audiovisual stimuli were selected because they are easier to implement in experimental settings and evoke stronger and more dynamic affective responses than static images or simple auditory cues [9]. Each video was designed to last approximately three to five minutes because excessively long stimuli can reduce both the validity of emotional induction and participants’ attentiveness [10].

### 3.3. Dataset

This study involved ten healthy adult participants (four males and six females; mean age = 28.1 ± 5.76 years), all of whom were Korean nationals with no history of medical conditions or use of psychotropic or cardiovascular medications. To ensure high-quality biosignal acquisition, we advised the participants to abstain from alcohol and obtain sufficient sleep the day prior to the experiment and avoid caffeine intake on the day of participation. Emotional video stimuli were viewed in an environment designed to minimize distractions and maximize immersion. To reduce carry-over effects and interference, participants rested for at least three minutes after each stimulus.

After each video stimulus, participants completed a questionnaire to assess their subjective emotional experiences. They rated the degree of emotional immersion and selected one or more emotions experienced from the categories of Joy, Disgust, Fear, Anger, Surprise, Sadness, and Other. When multiple emotions were selected, participants were instructed to assign relative weights to each emotion, ensuring a total score of 10, and to describe specific scenes or reasons that elicited these feelings. In addition, participants rated their emotional state along the valence, arousal, and dominance dimensions using a nine-point Likert scale. Valence indicated whether the stimulus was perceived as positive (closer to 9) or negative (closer to 1); arousal reflected whether the stimulus induced a calm (closer to 1) or excited (closer to 9) state; and dominance measured the extent of perceived control over the experienced emotion, with higher scores indicating greater control [11].

### 3.4. HRV Extraction

Participants wore a chest-strap sensor (Polar H10, Polar Electro, Kempele, Finland) while viewing the videos in a relaxed sitting posture at a distance of approximately 60 cm from the display monitor. From the continuous HRV data recorded during the experiment, two segments were extracted: a two-minute baseline segment measured before the stimulus during a resting state, and a two-minute segment recorded during the stimulus that captured the most emotion-relevant physiological response. The Polar H10 has been validated against laboratory-grade ECG systems, demonstrating accuracy, reliability, and reproducibility [12]. Its lightweight and flexible design minimized discomfort during data collection.

**Figure 1:**
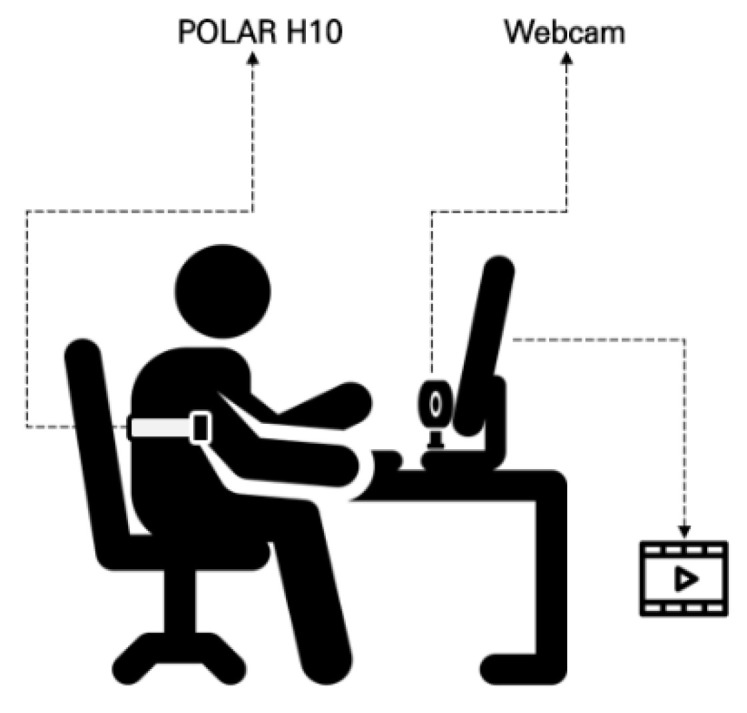
Overview of the experimental setup.

**Figure 2:**
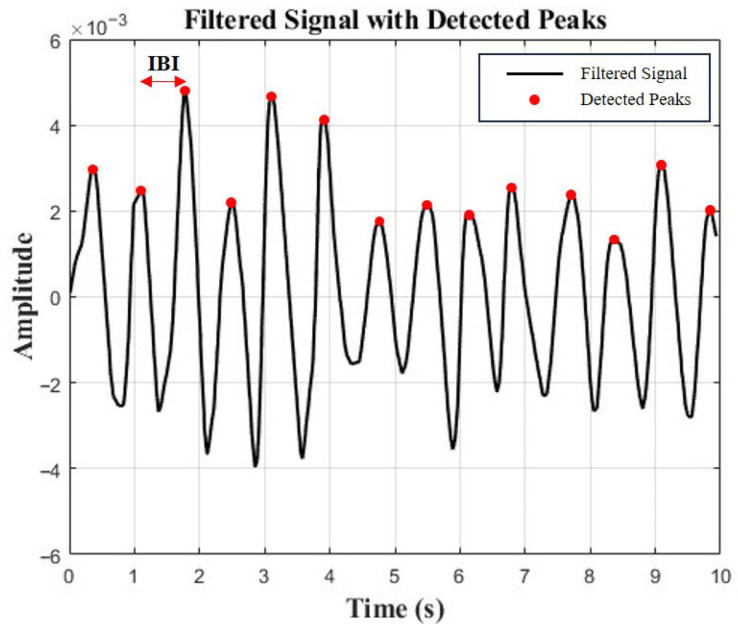
Peak detection and IBI calculation from rPPG signal.

To validate the effectiveness of the DHVS, a camera-based rPPG system, as an emotion recognition tool, HRV data were simultaneously collected using a webcam (C920 HD Pro, Logitech, Suzhou, China) and processed with the DHVS algorithm (Figure 1). The system was implemented in Python 3.12. Video input from the webcam was processed in real time with OpenCV, and facial regions were detected at approximately one-second intervals (30 frames) using a Haarcascade-based model. For each detected region of interest (ROI), the average intensity of the green channel was extracted frame by frame to generate the rPPG signal. Signals were accumulated in windows of ~300 frames, detrended using a moving average method, and bandpass filtered (0.7–3.0 Hz) with a Butterworth filter. A peak detection algorithm was then applied to identify heartbeats and compute inter-beat intervals (IBIs). Average IBIs were calculated, and their reciprocals yielded heart rate values. HRV parameters were subsequently derived from the extracted IBI sequences using the PyHRV library (version 0.4.1) (Figure 2).

HRV indices obtained from the Polar H10 and DHVS were synchronized to identical time intervals across each emotional segment (baseline–stimulation–recovery) and subsequently averaged at the condition level. This procedure was adopted to enable direct comparison between the two devices, as the contactless DHVS is relatively more sensitive to lighting and subject motion, which could lead to unequal effective signal lengths when shorter analysis windows are applied.

For analysis, relative changes were computed by comparing baseline (pre-stimulus) and during-stimulus data, rather than using absolute values. This approach accounts for individual differences in physiological states that could otherwise introduce variability in responses to identical stimuli.

(1) $$Rate\;of\;Change\left(\%\right)=\left(\frac{{HRV}_{during-stimulus}-{HRV}_{pre-stimulus}}{{HRV}_{pre-stimuluse}}\right)\;*\;100$$

### 3.5. HRV Feature Selection

The HRV indices selected for analysis, along with their definitions and characteristics, are summarized in the table (Table 2).

## 4. Results

### 4.1. Self-Reported VAD Scores

**Figure 3:**
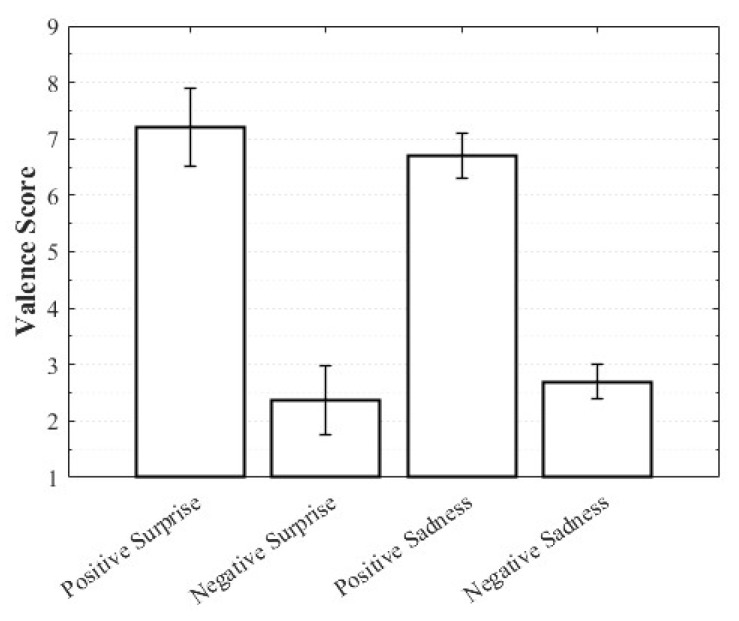
Valence score of complex emotions.

**Figure 4:**
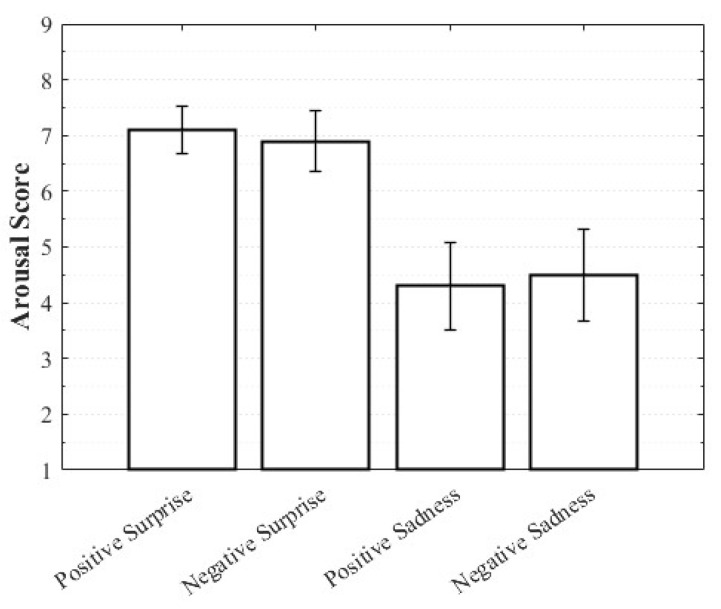
Arousal score of complex emotions.

**Figure 5:**
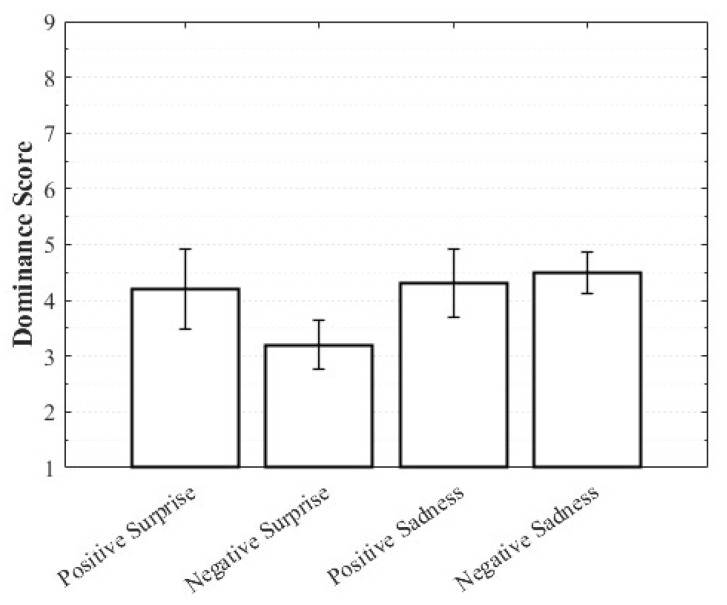
Dominance score of complex emotions.

Positive Surprise was characterized by relatively high valence and arousal with moderately low dominance, whereas Negative Surprise exhibited low valence, high arousal, and low dominance, yielding a profile similar to fear. Both Positive Sadness and Negative Sadness demonstrated similarly low arousal and dominance, but with opposite valence trends. These findings suggest that both emotions share a common subjective basis in sadness yet diverge along the valence dimension. The observation that VAD scores differed within the same emotional category depending on whether the stimulus was perceived as positive or negative highlights the complexity of emotions and the directional distinctions defined by valence (Figure 3, Figure 4 and Figure 5).

### 4.2. Emotion Proportions

**Figure 6:**
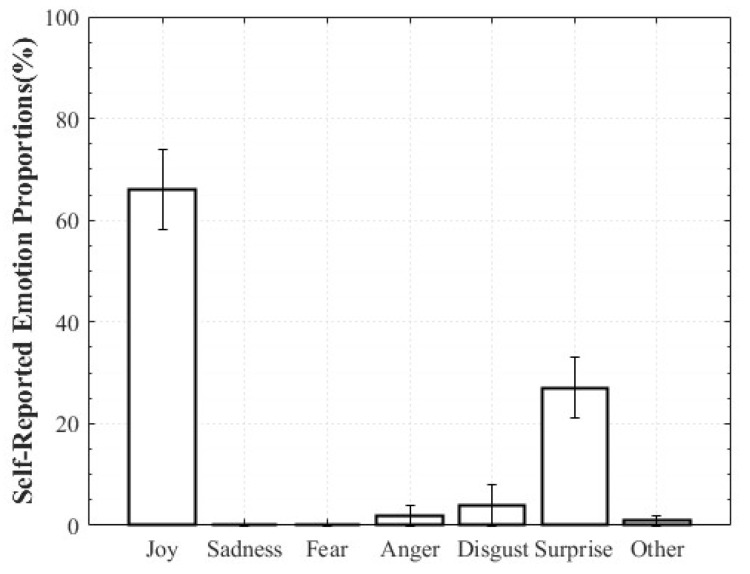
Reported Proportions of Positive Surprise.

**Figure 7:**
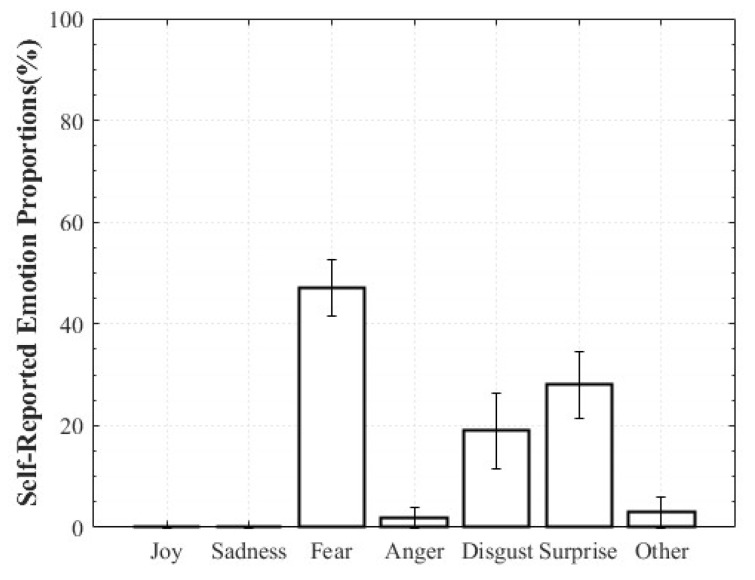
Reported Proportions of Negative Surprise.

**Figure 8:**
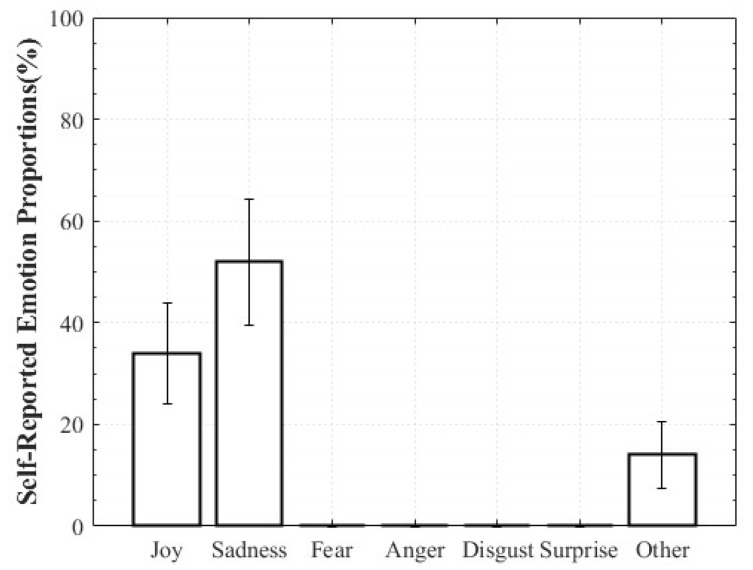
Reported Proportions of Positive Sadness.

**Figure 9:**
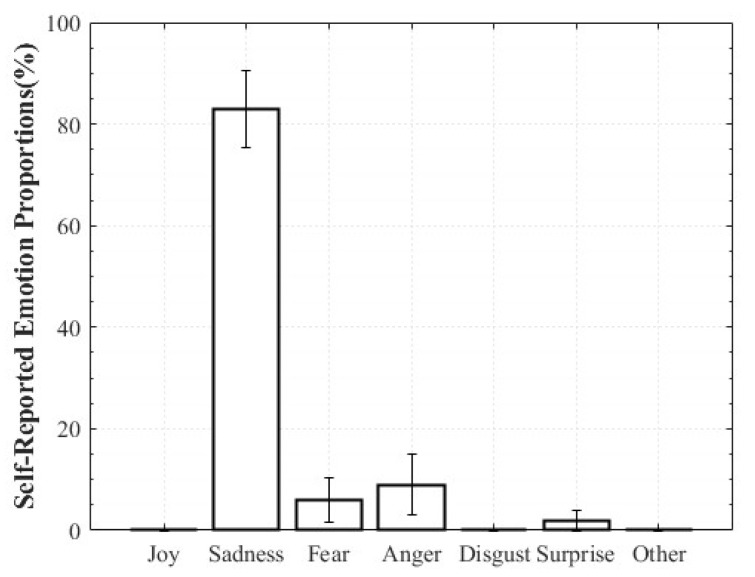
Reported Proportions of Negative Sadness.

For Positive Surprise, the reported composition was 27% Surprise and 66% Joy, indicating simultaneous activation of surprise and positive affect. Some participants additionally reported Fear and Disgust, explaining that the intensity of being startled led them to select those emotions. Negative Surprise was reported as 28% Surprise, 47% Fear, and 19% Disgust, suggesting that surprise co-occurred with multiple negative affective states. For Negative Sadness, participants reported on average 83% Sadness, 6% Fear, and 9% Anger, indicating that sadness was the dominant emotion accompanied by other negative emotions. In contrast, Positive Sadness elicited 52% Sadness and 34% Joy, reflecting the coexistence of theoretically opposing emotions. This pattern demonstrates that experiences described as “moving” can involve a blend of sadness and joy (Figure 6, Figure 7, Figure 8 and Figure 9). 

Overall, these results confirmed that complex emotions are not merely combinations of single discrete emotions but emerge through context-dependent interactions that produce nuanced affective states.

### 4.3. Statistical Significance of HRV Changes

To statistically examine the physiological differences in HRV indices measured under compound emotion stimuli, a one-sample *t*-test was performed to determine whether each index showed a significant change from zero within each emotion condition.

#### 4.3.1. Positive Surprise

In the Positive Surprise condition, Mean RR exhibited a non-significant trend toward an increase (*t*(9) = 1.847, *p* = 0.098, *M* = 2.12, *SD* = 3.62, *Cohen’s d* = 0.584), indicating a transient reduction in heart rate. This finding corresponded with previous reports that surprise is accompanied by decreased heart rate and elevated blood pressure [13].

#### 4.3.2. Negative Surprise

In the Negative Surprise condition, the mean RR increased significantly (*t*(8) = 3.079, *p* = 0.015, *M* = 7.79, *SD* = 7.59, *Cohen’s d* = 1.03) and Mean HR decreased significantly (*t*(8) = −3.298, *p* = 0.011, *M* = −6.84, *SD* = 6.22, *Cohen’s d* = −1.10), confirming that heart rate reduction is a consistent physiological response to surprise regardless of valence. Additionally, the SD1/SD2 ratio increased significantly (*t*(8) = 3.247, *p* = 0.012, *M* = 9.02, *SD* = 7.30, *Cohen’s d* = 1.30) suggesting potential autonomic imbalance due to parasympathetic predominance [14]. Surprise is known to involve a transient state of uncertainty immediately following the stimulus [15,16,17], which often interrupts ongoing thoughts and activities [18,19]. This combination of information processing inhibition, heightened arousal, and physiological suppression suggests a composite autonomic response that goes beyond a simple startle reflex and can instead be interpreted as passive freezing or cognitive blocking resulting from cognitive shock [20].

#### 4.3.3. Positive Sadness

For Positive Sadness, the total power decreased significantly (*t*(8) = −3.311, *p* = 0.011, *M* = −32.65, *SD* = 29.59, *Cohen’s d* = −1.10). Because total power reflects overall autonomic activity, this reduction indicates that Positive Sadness suppresses global autonomic responsiveness or limits physiological reactivity. Moreover, both SDNN, reflecting the overall heart rate variability (*t*(8) = −2.163, *p* = 0.062, *M* = −14.46, *SD* = 20.00, *Cohen’s d* = −0.723), and SD2, reflecting combined sympathetic–parasympathetic activity (*t*(8) = −2.043, *p* = 0.075, *M* = −13.09, *SD* = 19.22, *Cohen’s d* = −0.681), showed non-significant but decreasing trends. These results may reflect the immersive and empathic qualities of sadness, which dampen general autonomic activity [14].

#### 4.3.4. Negative Sadness

For Negative Sadness, overall HRV changes were observed, but no statistically significant differences were detected. Self-report evaluations indicated high levels of sadness, and although several participants exhibited decreases in long-term autonomic indices, large inter-individual variability precluded statistical significance. These findings may be attributed to differences in attentional regulation and emotion processing strategies among participants [21]. However, given the limited sample size, generalization is constrained, and further experimental validation in larger cohorts is warranted.

### 4.4. Correlation Between HRV Changes and Self-Reported Evaluations

#### 4.4.1. Positive Surprise

**Figure 10:**
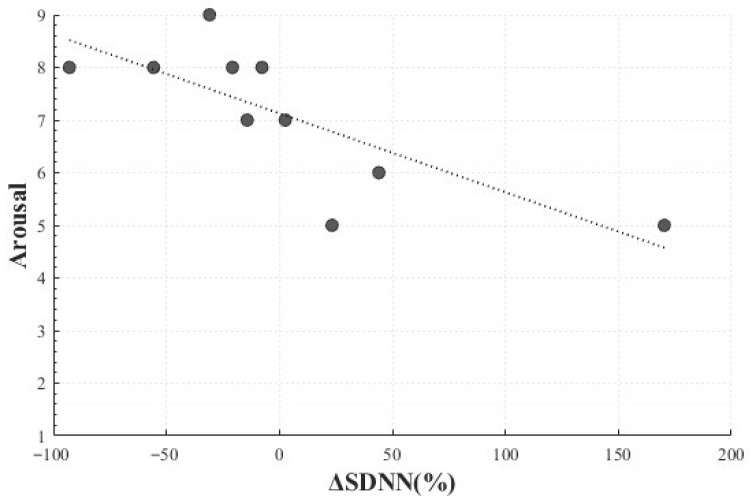
Correlation-Based Trend Line Between Relative Change in SDNN of Positive Surprise and Arousal.

In the Positive Surprise condition, a statistically significant negative linear correlation was observed between the change rate of SDNN (*M* = 1.81, *SD* = 70.58) and arousal (*M* = 7.10, *SD* = 1.37) (*r*(8) = −0.772, *p* = 0.009). This indicated that participants who reported higher levels of arousal tended to exhibit smaller changes in SDNN, suggesting that Positive Surprise may elicit heightened subjective arousal without necessarily producing a corresponding increase in autonomic responsiveness. In other words, emotional arousal may coexist with physiologically suppressed responses (Figure 10).

**Figure 11:**
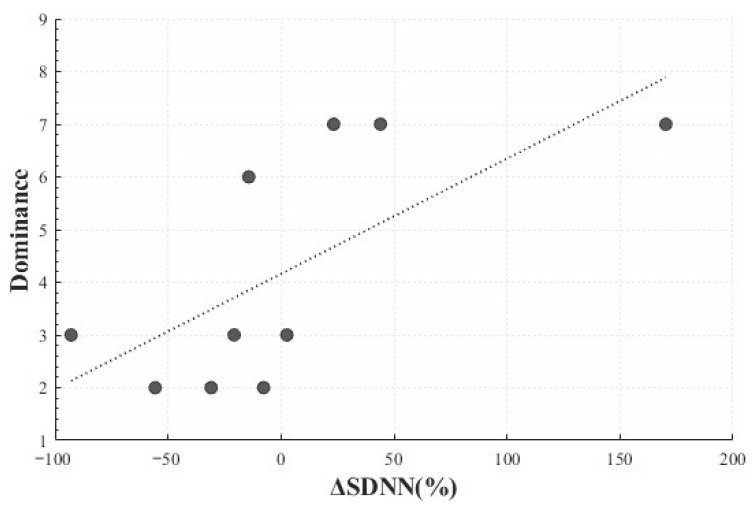
Correlation-Based Trend Line Between Relative Change in SDNN of Positive Surprise and Dominance.

A significant positive linear correlation was also observed between the SDNN change rate (*M* = 1.81, *SD* = 70.58) and dominance (*M* = 4.2, *SD* = 2.25) (*r*(8) = 0.686, *p* = 0.029), indicating that larger changes in SDNN were associated with greater perceived control. This finding suggests that differences in perceived control or regulation of surprise may be linked to autonomic responses (Figure 11).

**Figure 12:**
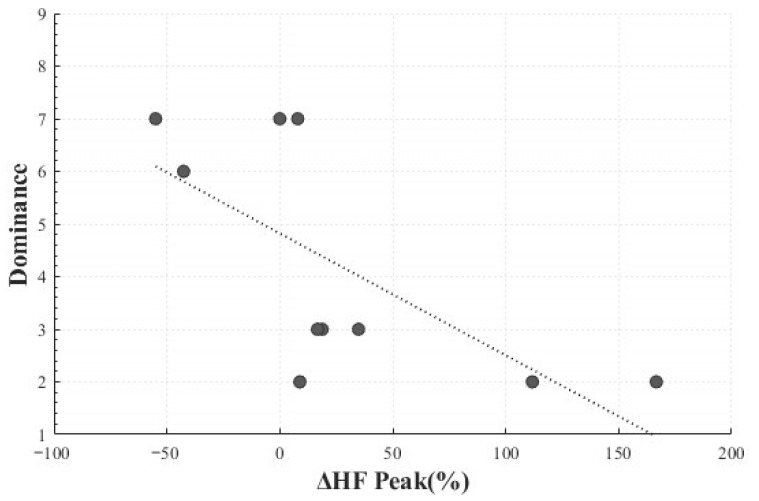
Correlation-Based Trend Line Between Relative Change in HF Peak of Positive Surprise and Dominance.

In addition, a significant negative correlation was identified between HF peak change rate (*M* = 26.79, *SD* = 66.59) and dominance (*M* = 4.2, *SD* = 2.25) (*r*(8) = −0.686, *p* = 0.029). This implies that greater HF peak changes corresponded to lower perceived control, suggesting that unexpected responses induced by Positive Surprise may involve parasympathetic activation associated with a reduced sense of control (Figure 12).

#### 4.4.2. Negative Surprise

In the Negative Surprise condition, a non-significant positive correlation was observed between the LF/HF change rate (*M* = −18.66, *SD* = 82.46) and valence (*M* = 2.78, *SD* = 1.99) (*r*(7) = 0.617, *p* = 0.058). This trend indicated that when parasympathetic activity was relatively dominant compared with sympathetic activity, participants tended to report higher valence.

#### 4.4.3. Positive Sadness

For Positive Sadness, no significant correlations were observed. This suggests that physiological responses to Positive Sadness were not strongly aligned with participants’ subjective reports. Possible explanations include missing HRV data from one participant, insufficient intensity or clarity of the stimulus, or the inherently complex nature of the emotion, which involves contradictory affective components. Because individual differences in context interpretation and immersion may further influence outcomes, these findings indicate the need for further validation in subsequent studies.

#### 4.4.4. Negative Sadness

**Figure 13:**
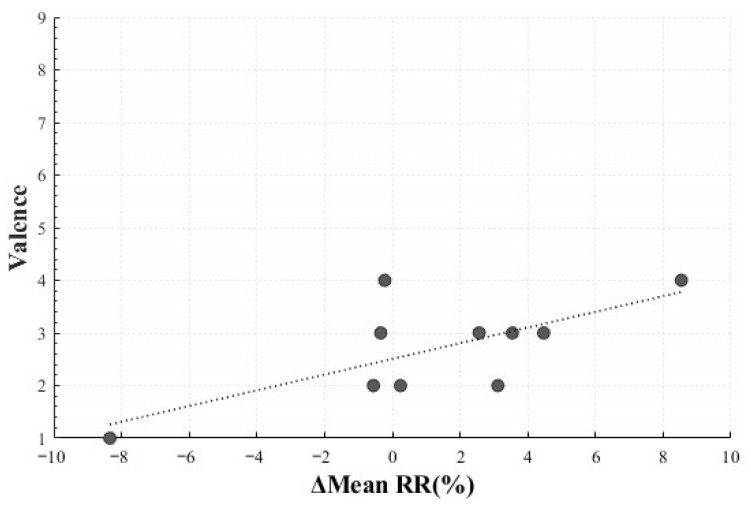
Correlation-Based Trend Line Between Relative Change in Mean RR of Negative Sadness and Valence.

In the Negative Sadness condition, a statistically significant positive correlation was observed between the mean RR change rate (*M* = 1.30, *SD* = 4.41) and valence (*M* = 2.70, *SD* = 0.95) (*r* = 0.693, *p* = 0.026). This result indicated that participants who exhibited greater increases in inter-beat intervals (reflecting heart rate reduction) tended to report higher valence. The stimulus for Negative Sadness—videos depicting bereaved families crying and being interviewed after tragic accidents—likely contributed to this effect. Rather than eliciting purely negative emotions, the stimulus may have invoked complex elements such as empathic engagement and immersion. Such psychological states may have manifested as parasympathetic dominance and increased inter-beat intervals, leading participants to report higher valence in connection with emotion regulation processes rooted in empathic involvement (Figure 13) [22].

### 4.5. HRV Responses by Complex Emotion

A repeated-measures analysis of variance (ANOVA) was conducted to examine changes in HRV indices for the four complex emotions defined in this study (Positive Surprise, Negative Surprise, Positive Sadness, and Negative Sadness). The independent variables were valence (positive/negative) and emotion (surprise/sadness), both treated as within-subject factors.

**Figure 14:**
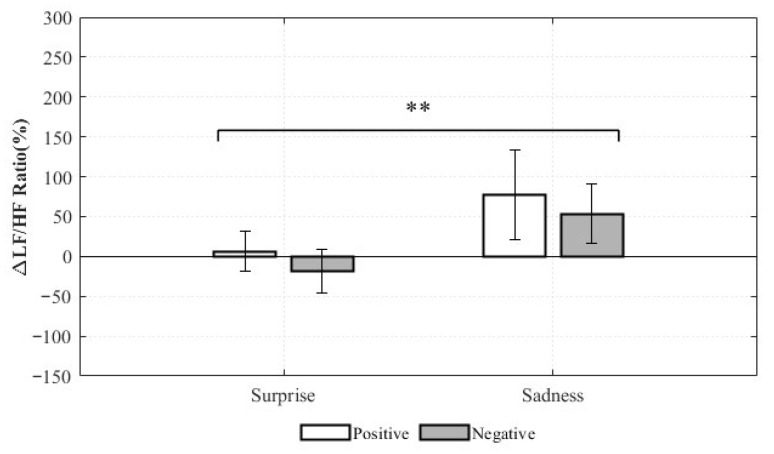
Relative Changes in LF/HF Ratio for Complex Emotions (** *p* < 0.01).

Significant effects were observed for the LF/HF ratio change rate and total power. For the LF/HF ratio, the main effect of valence was not statistically significant (*F*(1,8) = 0.318, *p* = 0.589, η*p*^2^ = 0.038), indicating no meaningful difference in LF/HF ratio changes between positive and negative valence within the same emotion category. This suggests that, within either Surprise or Sadness, the positive–negative distinction may not reliably differentiate the balance between sympathetic and parasympathetic activity. In contrast, the main effect of emotion was statistically significant (*F*(1,8) = 17.437, *p* = 0.003, η*p*^2^ = 0.686), demonstrating a clear difference in LF/HF ratio changes between Surprise and Sadness, irrespective of valence. As the LF/HF ratio typically reflects the relative balance of sympathetic and parasympathetic activity, these results suggest that Surprise and Sadness elicit distinct autonomic response patterns (Figure 14).

**Figure 15:**
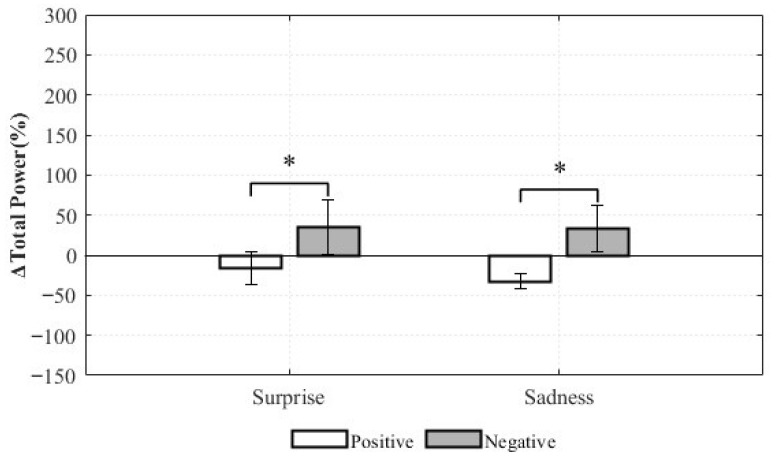
Relative Changes in Total power for Complex Emotions (* *p* < 0.05).

For the total power, the main effect of the valence was statistically significant (*F*(1,8) = 5.665, *p* = 0.045, η*p*^2^ = 0.415). This indicates that, regardless of emotion type, negative-valence stimuli had a greater impact on overall autonomic responsiveness than positive-valence stimuli. This finding supports the notion that negative emotions may induce stronger physiological imbalances than positive emotions. In contrast, the main effect of Emotion was not statistically significant (*F*(1,8) = 0.111, *p* = 0.747, η*p*^2^ = 0.014), indicating that the distinction between Surprise and Sadness did not significantly influence total power changes (Figure 15).

These findings suggest that the LF/HF ratio is more sensitive to emotion type (Surprise vs. Sadness), while Total Power is more sensitive to valence (positive vs. negative). Thus, HRV indices reflect different dimensions of emotional composition: LF/HF ratio emphasizes differences in autonomic balance across emotion categories, whereas Total Power highlights differences in global autonomic reactivity across valence. These results underscore the need for careful selection and nuanced interpretation of HRV indices in emotion recognition research, taking into account the distinct physiological sensitivities of each measure.

### 4.6. Concordance Between DHVS and Polar H10

**Figure 16:**
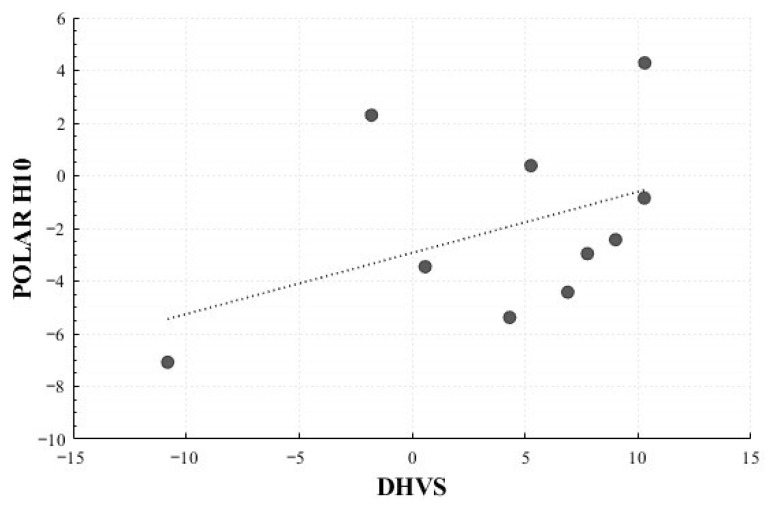
Correlation-Based Trend Line Between Relative Change in Mean HR of Positive Surprise: DHVS vs. Polar H10.

**Figure 17:**
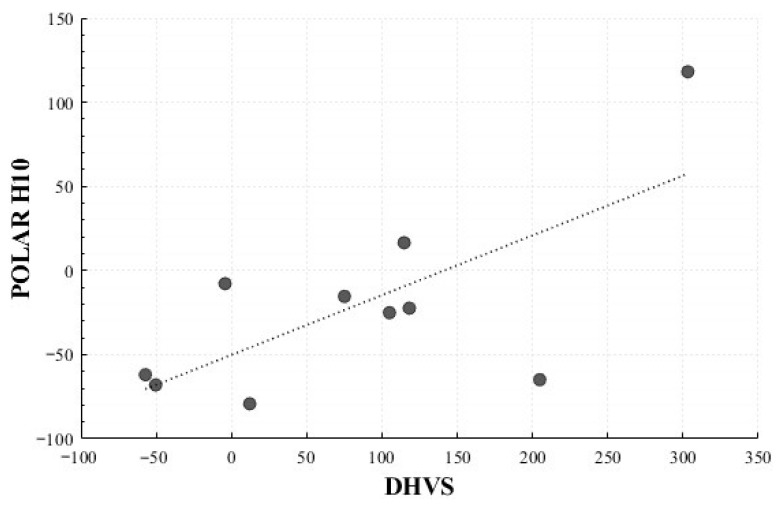
Correlation-Based Trend Line Between Relative Change in Total Power of Positive Surprise: DHVS vs. Polar H10.

In the Positive Surprise condition, significant correlations were observed for the mean HR change rate (*r* = 0.716, *p* = 0.020) and total power change rate (*r* = 0.710, *p* = 0.021). In the Positive Sadness condition, a non-significant trend was observed for LF Peak change rate (*r* = 0.619, *p* = 0.102). No significant correlations were identified in the Negative Surprise or Negative Sadness conditions (Figure 16 and Figure 17).

## 5. Discussion

In this study, four compound emotions—Positive Surprise, Negative Surprise, Positive Sadness, and Negative Sadness—were defined by valence direction within basic emotion categories. We examined whether these states can be quantitatively identified from HRV-based physiological responses and how they align with self-reported VAD evaluations, to inform vision-based, contactless emotion recognition.

Significant HRV responses were observed for Positive Surprise, Negative Surprise, and Positive Sadness. Negative Surprise elicited a combined autonomic pattern with decreased heart rate and increased SD1/SD2, indicating inhibition; Positive Sadness showed a significant decrease in total power, suggesting reduced autonomic activation potentially linked to emotional immersion/empathy. These findings indicate that complex emotions exhibit distinct physiological profiles shaped by valence and cognitive processing.

Correlational analyses showed meaningful VAD–HRV links. In Positive Surprise, SDNN change correlated negatively with Arousal, consistent with emotionally heightened yet physiologically moderated responses. Associations between SDNN/HF Peak and Dominance suggest that perceived control over surprising stimuli relates to parasympathetic regulation. In Negative Sadness, mean RR change correlated positively with Valence, consistent with parasympathetic-dominant responses under empathic engagement.

The separation of each emotion condition into positive and negative valence within the same basic emotion was intended to experimentally reflect affective–ecological differences that are actually observed in everyday contexts. However, because HRV fundamentally indexes the level of autonomic nervous system activation, it should also be noted that in this study the associations were observed more consistently with arousal and, to a partial extent, with dominance, rather than with the discrete emotions themselves.

The repeated-measures ANOVA revealed that LF/HF ratio changes were more sensitive to emotion category (Surprise vs. Sadness), whereas total power changes were more sensitive to valence (positive vs. negative). This suggests that the LF/HF ratio is better suited to distinguishing between types of emotional responses, while total power is more effective in differentiating positive engagement from negative arousal. These findings highlight the importance of selecting appropriate HRV indicators based on the classification objective in HRV-based emotion models. However, given the limited sample size, these patterns should be interpreted as preliminary, taking into account both statistical significance and effect size.

The valence-based separation of emotion conditions within basic emotion categories was intended to experimentally reflect ecological differences in affective experience observed in everyday contexts. Since HRV primarily reflects autonomic activation rather than emotion per se, the current results also suggest that arousal and dominance may be more stable physiological correlates than valence itself.

Furthermore, this study directly compared the contactless DHVS with the chest-strap reference device (Polar H10) under identical emotion-induction conditions. By extracting the same set of HRV indices as the reference device, we assessed the extent to which an rPPG-based contactless system can be harmonized with the HRV analytic framework commonly used in clinical research. Because DHVS minimizes interference from contact sensors and enables HRV acquisition in more naturalistic conditions, it satisfies a key prerequisite for future emotion monitoring in everyday environments.

A correlation analysis revealed condition-dependent consistency between the two devices. The Positive Surprise condition showed significant concordance for mean HR and total power, while the Positive Sadness condition exhibited a non-significant but notable trend for LF peak. In contrast, no significant concordance was observed for Negative Surprise or Negative Sadness, possibly due to the lower sensitivity of DHVS to subtle autonomic changes or interference from external factors such as lighting and participant movement. These findings indicate that although DHVS can capture autonomic responses to a certain extent, further refinement is required to reduce external noise and better capture physiological sensitivity across different emotional contexts. Therefore, these results should be interpreted as preliminary.

Overall, these findings suggest that DHVS can partially reflect autonomic nervous system responses. Moreover, rather than applying a single indicator uniformly, emotion recognition systems should select indices tailored to emotion-specific physiological patterns and to the characteristics of each sensing modality. Through such developments, DHVS has the potential to evolve into a contactless, biosignal-based emotion recognition system capable of real-time emotion identification across diverse environments, with broad applicability in healthcare, affective computing, and human–machine interaction.

Additionally, the correlation matrices presented in Supplementary Tables S1–S8 revealed that HRV indices obtained from both Polar H10 and DHVS exhibited highly similar clustering structures. The consistent grouping of key time-domain indices and total power across both devices supports the feasibility of using rPPG-based HRV measures as representative physiological features for emotion recognition.

## 6. Conclusions

This study empirically demonstrated that complex emotions are not mere combinations of basic emotions but are accompanied by independent and multidimensional physiological responses. Moreover, it established quantitative associations between subjective reports of emotional experience and corresponding physiological responses, thereby supporting the feasibility of biosignal-based emotion recognition that reflects the complexity of affective states.

Because this analysis involved repeated testing of multiple HRV indices across emotional conditions, the increased number of comparisons may have raised the risk of a Type I error. Therefore, the present results should be interpreted at an exploratory level. Future studies should apply multivariate approaches—such as false discovery rate (FDR) correction or principal component analysis (PCA)—to systematically reduce the dimensionality of HRV indicators.

This study was conducted with a relatively small number of participants, and the validation of stimulus adequacy was limited, which restricts the generalizability of the findings. In addition, since condition-level averages were used, intra-individual and within-condition variations could not be modeled. As both sensors provide heartbeat-level time series, future studies should apply within-subject analytical techniques such as mixed-effects modeling to capture more detailed temporal patterns of emotional responses. Furthermore, it will be necessary to refine the emotion-inducing stimuli and verify their effectiveness to ensure validity. Expanding the number of participants will also enhance statistical power and help control individual differences, thereby improving the accuracy and reliability of emotion-specific responses. Ultimately, these advancements are expected to contribute to the development of high-dimensional artificial intelligence systems capable of recognizing complex human emotions.

## Figures and Tables

**Table 1:** Developed emotional stimuli.

Emotion	Content
Positive Surprise	Positive scenes incorporating jump-scare techniques
Negative Surprise	Sudden appearance of a ghost in a tense atmosphere
Positive Sadness	Children crying while speaking with their parents by phone
Negative Sadness	Bereaved families mourning the loss of children in a disaster

**Table 2:** HRV features and definitions.

Feature	Unit	Definition
MEAN RR	ms	Mean of RR intervals
SDNN	ms	The standard deviation of Normal-to-Normal (NN) Intervals
RMSSD	ms	Root mean square of successive RR interval differences
SD1	ms	Poincaré plot standard deviation perpendicular the line of identity
SD2	ms	Poincaré plot standard deviation along the line of identity
SD1/SD2		The ratio of SD1-to-SD2 frequency
LF	ms^2^	Low-frequency band (0.04–0.15 Hz)
HF	ms^2^	High-frequency band (0.15–0.4 Hz)
LF/HF		The ratio of LF-to-HF frequency
TOTAL POWER	ms^2^	The sum of spectral power in the VLF, LF, and HF bands

## Data Availability

The datasets presented in this article are not readily available due to limitations in sample size. Requests for access to the datasets should be directed to the corresponding author.

## Supplementary Materials

The following supporting information can be downloaded at https://www.mdpi.com/article/10.3390/healthcare13233036/s1.
