# Supplementary material for: HRV-Based Recognition of Complex Emotions: Feature Identification and Emotion-Specific Indicator Selection
Source: Healthcare (Basel). 2025 Nov 24;13(23):3036. doi: 10.3390/healthcare13233036 (PMC12692675; doi:10.3390/healthcare13233036)
Supplement: Supplementary file 1 [file healthcare-13-03036-s001.zip › healthcare-3930228-supplementary.pdf]

Table S1. Polar H10-based HRV correlation matrix: Positive surprise

		Mean RR	SDNN	Mean HR	RMSSD	LF peak	HF peak	LF power	HF power	LFtoHF ratio	Total power	SD1	SD2	SD1to SD2 ratio
**Mean RR**	**Pearson correlation**	1	0.476	-.999**	0.513	0.284	0.229	0.199	0.535	-0.431	0.421	0.513	0.414	0.405
	**Two-tailed p-value**		0.164	0.000	0.130	0.426	0.525	0.582	0.111	0.213	0.226	0.130	0.234	0.246
	**N**	10	10	10	10	10	10	10	10	10	10	10	10	10
**SDNN**	**Pearson correlation**	0.476	1	-0.481	.915**	-0.258	-0.349	0.553	.810**	-0.116	.782**	.915**	.967**	0.522
	**Two-tailed p-value**	0.164		0.159	0.000	0.471	0.324	0.097	0.004	0.750	0.008	0.000	0.000	0.121
	**N**	10	10	10	10	10	10	10	10	10	10	10	10	10
**Mean HR**	**Pearson correlation**	-.999**	-0.481	1	-0.515	-0.292	-0.220	-0.185	-0.534	0.443	-0.409	-0.515	-0.422	-0.391
	**Two-tailed p-value**	0.000	0.159		0.128	0.413	0.541	0.608	0.112	0.199	0.240	0.128	0.225	0.264
	**N**	10	10	10	10	10	10	10	10	10	10	10	10	10
**RMSSD**	**Pearson correlation**	0.513	.915**	-0.515	1	-0.038	-0.167	0.426	.958**	-0.379	.804**	1.000**	.785**	0.612
	**Two-tailed p-value**	0.130	0.000	0.128		0.917	0.645	0.219	0.000	0.280	0.005	0.000	0.007	0.060
	**N**	10	10	10	10	10	10	10	10	10	10	10	10	10
**LF peak**	**Pearson correlation**	0.284	-0.258	-0.292	-0.038	1	0.413	-0.369	0.076	-0.587	-0.236	-0.038	-0.340	-0.018
	**Two-tailed p-value**	0.426	0.471	0.413	0.917		0.236	0.295	0.835	0.075	0.511	0.916	0.337	0.960
	**N**	10	10	10	10	10	10	10	10	10	10	10	10	10
**HF peak**	**Pearson correlation**	0.229	-0.349	-0.220	-0.167	0.413	1	-0.087	-0.198	0.017	-0.124	-0.167	-0.410	-0.004

	**Two-tailed p-value**	0.525	0.324	0.541	0.645	0.236		0.812	0.583	0.964	0.732	0.645	0.239	0.992
	**N**	10	10	10	10	10	10	10	10	10	10	10	10	10
**LF power**	**Pearson correlation**	0.199	0.553	-0.185	0.426	-0.369	-0.087	1	0.391	0.504	.832**	0.426	0.572	0.553
	**Two-tailed p-value**	0.582	0.097	0.608	0.219	0.295	0.812		0.263	0.138	0.003	0.220	0.084	0.097
	**N**	10	10	10	10	10	10	10	10	10	10	10	10	10
**HF power**	**Pearson correlation**	0.535	.810**	-0.534	.958**	0.076	-0.198	0.391	1	-0.493	.801**	.958**	.645*	.646*
	**Two-tailed p-value**	0.111	0.004	0.112	0.000	0.835	0.583	0.263		0.147	0.005	0.000	0.044	0.044
	**N**	10	10	10	10	10	10	10	10	10	10	10	10	10
**LFto HF ratio**	**Pearson correlation**	-0.431	-0.116	0.443	-0.379	-0.587	0.017	0.504	-0.493	1	0.022	-0.379	0.052	-0.230
	**Two-tailed p-value**	0.213	0.750	0.199	0.280	0.075	0.964	0.138	0.147		0.951	0.280	0.886	0.523
	**N**	10	10	10	10	10	10	10	10	10	10	10	10	10
**Total power**	**Pearson correlation**	0.421	.782**	-0.409	.804**	-0.236	-0.124	.832**	.801**	0.022	1	.804**	.683*	.759*
	**Two-tailed p-value**	0.226	0.008	0.240	0.005	0.511	0.732	0.003	0.005	0.951		0.005	0.030	0.011
	**N**	10	10	10	10	10	10	10	10	10	10	10	10	10
**SD1**	**Pearson correlation**	0.513	.915**	-0.515	1.000**	-0.038	-0.167	0.426	.958**	-0.379	.804**	1	.785**	0.612
	**Two-tailed p-value**	0.130	0.000	0.128	0.000	0.916	0.645	0.220	0.000	0.280	0.005		0.007	0.060
	**N**	10	10	10	10	10	10	10	10	10	10	10	10	10
**SD2**	**Pearson correlation**	0.414	.967**	-0.422	.785**	-0.340	-0.410	0.572	.645*	0.052	.683*	.785**	1	0.387

	**Two-tailed p-value**	0.234	0.000	0.225	0.007	0.337	0.239	0.084	0.044	0.886	0.030	0.007		0.269
	**N**	10	10	10	10	10	10	10	10	10	10	10	10	10
**SD1 to SD2 ratio**	**Pearson correlation**	0.405	0.522	-0.391	0.612	-0.018	-0.004	0.553	.646*	-0.230	.759*	0.612	0.387	1
	**Two-tailed p-value**	0.246	0.121	0.264	0.060	0.960	0.992	0.097	0.044	0.523	0.011	0.060	0.269	
	**N**	10	10	10	10	10	10	10	10	10	10	10	10	10

**. Correlation is significant at the 0.01 level (Two-tailed).

*. Correlation is significant at the 0.05 level (Two-tailed).

Table S2. Polar H10-based HRV correlation matrix: Positive sadness

		Mean RR	SDNN	Mean HR	RMSSD	LF peak	HF peak	LF power	HF power	LFtoHF ratio	Total power	SD1	SD2	SD1to SD2 ratio
Mean RR	Pearson correlation	1	0.614	-1.000**	.781*	0.438	0.285	-0.041	0.445	-0.179	0.035	.781*	0.518	-0.438
	Two-tailed p-value		0.105	0.000	0.022	0.278	0.494	0.923	0.270	0.671	0.934	0.022	0.189	0.278
	N	8	8	8	8	8	8	8	8	8	8	8	8	8
SDNN	Pearson correlation	0.614	1	-0.615	0.652	0.130	-0.241	0.433	0.155	0.351	0.333	0.652	.962**	-0.170
	Two-tailed p-value	0.105		0.105	0.080	0.758	0.565	0.284	0.715	0.394	0.420	0.080	0.000	0.688
	N	8	8	8	8	8	8	8	8	8	8	8	8	8
Mean HR	Pearson correlation	-1.000**	-0.615	1	-.781*	-0.432	-0.268	0.058	-0.450	0.194	-0.029	-.782*	-0.517	0.430
	Two-tailed p-value	0.000	0.105		0.022	0.285	0.522	0.892	0.263	0.645	0.946	0.022	0.190	0.287
	N	8	8	8	8	8	8	8	8	8	8	8	8	8
RMSSD	Pearson correlation	.781*	0.652	-.781*	1	0.524	0.360	-0.137	0.664	-0.325	0.219	1.000**	0.442	-0.371
	Two-tailed p-value	0.022	0.080	0.022		0.183	0.381	0.747	0.072	0.431	0.602	0.000	0.273	0.366
	N	8	8	8	8	8	8	8	8	8	8	8	8	8
LF peak	Pearson correlation	0.438	0.130	-0.432	0.524	1	0.679	-0.381	0.174	-0.165	-0.588	0.524	0.046	-0.035
	Two-tailed p-value	0.278	0.758	0.285	0.183		0.064	0.351	0.681	0.696	0.126	0.183	0.914	0.935
	N	8	8	8	8	8	8	8	8	8	8	8	8	8

**HF peak**	**Pearson correlation**	0.285	-0.241	-0.268	0.360	0.679	1	-0.160	0.253	-0.206	-0.152	0.360	-0.337	-0.361
	**Two-tailed p-value**	0.494	0.565	0.522	0.381	0.064		0.705	0.546	0.624	0.719	0.381	0.414	0.380
	**N**	8	8	8	8	8	8	8	8	8	8	8	8	8
**LF power**	**Pearson correlation**	-0.041	0.433	0.058	-0.137	-0.381	-0.160	1	-0.355	.877**	0.594	-0.137	0.574	-0.210
	**Two-tailed p-value**	0.923	0.284	0.892	0.747	0.351	0.705		0.388	0.004	0.120	0.747	0.137	0.617
	**N**	8	8	8	8	8	8	8	8	8	8	8	8	8
**HF power**	**Pearson correlation**	0.445	0.155	-0.450	0.664	0.174	0.253	-0.355	1	-0.653	0.345	0.664	-0.060	-0.672
	**Two-tailed p-value**	0.270	0.715	0.263	0.072	0.681	0.546	0.388		0.079	0.403	0.072	0.888	0.068
	**N**	8	8	8	8	8	8	8	8	8	8	8	8	8
**LFto HF ratio**	**Pearson correlation**	-0.179	0.351	0.194	-0.325	-0.165	-0.206	.877**	-0.653	1	0.160	-0.325	0.558	0.094
	**Two-tailed p-value**	0.671	0.394	0.645	0.431	0.696	0.624	0.004	0.079		0.705	0.432	0.151	0.824
	**N**	8	8	8	8	8	8	8	8	8	8	8	8	8
**Total power**	**Pearson correlation**	0.035	0.333	-0.029	0.219	-0.588	-0.152	0.594	0.345	0.160	1	0.219	0.273	-0.459
	**Two-tailed p-value**	0.934	0.420	0.946	0.602	0.126	0.719	0.120	0.403	0.705		0.602	0.513	0.253
	**N**	8	8	8	8	8	8	8	8	8	8	8	8	8
**SD1**	**Pearson correlation**	.781*	0.652	-.782*	1.000**	0.524	0.360	-0.137	0.664	-0.325	0.219	1	0.442	-0.371
	**Two-tailed p-value**	0.022	0.080	0.022	0.000	0.183	0.381	0.747	0.072	0.432	0.602		0.273	0.366
	**N**	8	8	8	8	8	8	8	8	8	8	8	8	8

**SD2**	**Pearson correlation**	0.518	.962**	-0.517	0.442	0.046	-0.337	0.574	-0.060	0.558	0.273	0.442	1	-0.096
	**Two-tailed p-value**	0.189	0.000	0.190	0.273	0.914	0.414	0.137	0.888	0.151	0.513	0.273		0.822
	**N**	8	8	8	8	8	8	8	8	8	8	8	8	8
**SD1 to SD2 ratio**	**Pearson correlation**	-0.438	-0.170	0.430	-0.371	-0.035	-0.361	-0.210	-0.672	0.094	-0.459	-0.371	-0.096	1
	**Two-tailed p-value**	0.278	0.688	0.287	0.366	0.935	0.380	0.617	0.068	0.824	0.253	0.366	0.822	
	**N**	8	8	8	8	8	8	8	8	8	8	8	8	8

**. Correlation is significant at the 0.01 level (Two-tailed).

*. Correlation is significant at the 0.05 level (Two-tailed).

Table S3. Polar H10-based HRV correlation matrix: Negative surprise

		Mean RR	SDNN	Mean HR	RMSSD	LF peak	HF peak	LF power	HF power	LFtoHF ratio	Total power	SD1	SD2	SD1to SD2 ratio
Mean RR	Pearson correlation	1	.799**	-.996**	.714*	0.628	-0.651	0.321	0.578	-0.224	0.659	.714*	.708*	-0.029
	Two-tailed p-value		0.010	0.000	0.031	0.070	0.057	0.400	0.103	0.562	0.053	0.031	0.033	0.940
	N	9	9	9	9	9	9	9	9	9	9	9	9	9
SDNN	Pearson correlation	.799**	1	-.774*	.824**	.703*	-.796*	.734*	.798*	0.057	.944**	.824**	.978**	0.107
	Two-tailed p-value	0.010		0.014	0.006	0.035	0.010	0.024	0.010	0.884	0.000	0.006	0.000	0.784
	N	9	9	9	9	9	9	9	9	9	9	9	9	9
Mean HR	Pearson correlation	-.996**	-.774*	1	-.692*	-0.599	0.632	-0.300	-0.550	0.218	-0.621	-.692*	-.689*	0.010
	Two-tailed p-value	0.000	0.014		0.039	0.088	0.068	0.433	0.125	0.573	0.074	0.039	0.040	0.979
	N	9	9	9	9	9	9	9	9	9	9	9	9	9
RMSSD	Pearson correlation	.714*	.824**	-.692*	1	.851**	-.893**	0.468	.945**	-0.377	.827**	1.000**	.733*	0.509
	Two-tailed p-value	0.031	0.006	0.039		0.004	0.001	0.204	0.000	0.317	0.006	0.000	0.025	0.162
	N	9	9	9	9	9	9	9	9	9	9	9	9	9
LF peak	Pearson correlation	0.628	.703*	-0.599	.851**	1	-.840**	0.512	.689*	-0.148	.761*	.852**	0.607	0.475
	Two-tailed p-value	0.070	0.035	0.088	0.004		0.005	0.159	0.040	0.704	0.017	0.004	0.083	0.196
	N	9	9	9	9	9	9	9	9	9	9	9	9	9
HF peak	Pearson correlation	-0.651	-.796*	0.632	-.893**	-.840**	1	-0.499	-.844**	0.215	-.811**	-.893**	-.715*	-0.401

	**Two-tailed p-value**	0.057	0.010	0.068	0.001	0.005		0.171	0.004	0.579	0.008	0.001	0.030	0.285
	**N**	9	9	9	9	9	9	9	9	9	9	9	9	9
**LF power**	**Pearson correlation**	0.321	.734*	-0.300	0.468	0.512	-0.499	1	0.477	0.605	.831**	0.468	.786*	0.121
	**Two-tailed p-value**	0.400	0.024	0.433	0.204	0.159	0.171		0.194	0.084	0.005	0.204	0.012	0.757
	**N**	9	9	9	9	9	9	9	9	9	9	9	9	9
**HF power**	**Pearson correlation**	0.578	.798*	-0.550	.945**	.689*	-.844**	0.477	1	-0.391	.829**	.945**	.725*	0.410
	**Two-tailed p-value**	0.103	0.010	0.125	0.000	0.040	0.004	0.194		0.299	0.006	0.000	0.027	0.273
	**N**	9	9	9	9	9	9	9	9	9	9	9	9	9
**LFto HF ratio**	**Pearson correlation**	-0.224	0.057	0.218	-0.377	-0.148	0.215	0.605	-0.391	1	0.106	-0.377	0.197	-0.229
	**Two-tailed p-value**	0.562	0.884	0.573	0.317	0.704	0.579	0.084	0.299		0.786	0.317	0.612	0.554
	**N**	9	9	9	9	9	9	9	9	9	9	9	9	9
**Total power**	**Pearson correlation**	0.659	.944**	-0.621	.827**	.761*	-.811**	.831**	.829**	0.106	1	.827**	.903**	0.155
	**Two-tailed p-value**	0.053	0.000	0.074	0.006	0.017	0.008	0.005	0.006	0.786		0.006	0.001	0.690
	**N**	9	9	9	9	9	9	9	9	9	9	9	9	9
**SD1**	**Pearson correlation**	.714*	.824**	-.692*	1.000**	.852**	-.893**	0.468	.945**	-0.377	.827**	1	.734*	0.509
	**Two-tailed p-value**	0.031	0.006	0.039	0.000	0.004	0.001	0.204	0.000	0.317	0.006		0.024	0.162
	**N**	9	9	9	9	9	9	9	9	9	9	9	9	9
**SD2**	**Pearson correlation**	.708*	.978**	-.689*	.733*	0.607	-.715*	.786*	.725*	0.197	.903**	.734*	1	0.102

	**Two-tailed p-value**	0.033	0.000	0.040	0.025	0.083	0.030	0.012	0.027	0.612	0.001	0.024		0.793
	**N**	9	9	9	9	9	9	9	9	9	9	9	9	9
**SD1 to SD2 ratio**	**Pearson correlation**	-0.029	0.107	0.010	0.509	0.475	-0.401	0.121	0.410	-0.229	0.155	0.509	0.102	1
	**Two-tailed p-value**	0.940	0.784	0.979	0.162	0.196	0.285	0.757	0.273	0.554	0.690	0.162	0.793	
	**N**	9	9	9	9	9	9	9	9	9	9	9	9	9

**. Correlation is significant at the 0.01 level (Two-tailed).

*. Correlation is significant at the 0.05 level (Two-tailed).

Table S4. Polar H10-based HRV correlation matrix: Negative sadness

		Mean RR	SDNN	Mean HR	RMSSD	LF peak	HF peak	LF power	HF power	LFtoHF ratio	Total power	SD1	SD2	SD1to SD2 ratio
**Mean RR**	**Pearson correlation**	1	.645*	-.998**	.804**	0.308	-0.438	0.611	.647*	0.141	.656*	.805**	0.502	0.309
	**Two-tailed p-value**		0.044	0.000	0.005	0.387	0.205	0.061	0.043	0.697	0.039	0.005	0.139	0.385
	**N**	10	10	10	10	10	10	10	10	10	10	10	10	10
**SDNN**	**Pearson correlation**	.645*	1	-.641*	0.572	0.433	-0.317	0.580	0.269	0.433	0.286	0.572	.959**	0.261
	**Two-tailed p-value**	0.044		0.046	0.084	0.211	0.373	0.079	0.452	0.211	0.423	0.084	0.000	0.467
	**N**	10	10	10	10	10	10	10	10	10	10	10	10	10
**Mean HR**	**Pearson correlation**	-.998**	-.641*	1	-.793**	-0.302	0.466	-0.591	-.637*	-0.137	-0.632	-.794**	-0.501	-0.292
	**Two-tailed p-value**	0.000	0.046		0.006	0.396	0.175	0.072	0.047	0.705	0.050	0.006	0.140	0.412
	**N**	10	10	10	10	10	10	10	10	10	10	10	10	10
**RMSSD**	**Pearson correlation**	.804**	0.572	-.793**	1	.691*	0.000	0.421	.837**	0.023	0.319	1.000**	0.362	0.164
	**Two-tailed p-value**	0.005	0.084	0.006		0.027	1.000	0.225	0.002	0.950	0.369	0.000	0.304	0.651
	**N**	10	10	10	10	10	10	10	10	10	10	10	10	10
**LF peak**	**Pearson correlation**	0.308	0.433	-0.302	.691*	1	0.120	0.409	0.414	0.398	0.041	.690*	0.287	0.004
	**Two-tailed p-value**	0.387	0.211	0.396	0.027		0.740	0.241	0.234	0.254	0.911	0.027	0.422	0.991
	**N**	10	10	10	10	10	10	10	10	10	10	10	10	10
**HF peak**	**Pearson correlation**	-0.438	-0.317	0.466	0.000	0.120	1	-0.232	0.021	-0.206	-0.307	0.000	-0.349	-0.194

	Two-tailed p-value	0.205	0.373	0.175	1.000	0.740		0.519	0.954	0.569	0.389	1.000	0.323	0.590
	N	10	10	10	10	10	10	10	10	10	10	10	10	10
LFpower	Pearson correlation	0.611	0.580	-0.591	0.421	0.409	-0.232	1	0.063	.808**	.785**	0.422	0.525	0.349
	Two-tailed p-value	0.061	0.079	0.072	0.225	0.241	0.519		0.863	0.005	0.007	0.225	0.119	0.324
	N	10	10	10	10	10	10	10	10	10	10	10	10	10
HF power	Pearson correlation	.647*	0.269	-.637*	.837**	0.414	0.021	0.063	1	-0.388	0.219	.837**	0.129	-0.156
	Two-tailed p-value	0.043	0.452	0.047	0.002	0.234	0.954	0.863		0.268	0.544	0.003	0.723	0.667
	N	10	10	10	10	10	10	10	10	10	10	10	10	10
LFto HFratio	Pearson correlation	0.141	0.433	-0.137	0.023	0.398	-0.206	.808**	-0.388	1	0.368	0.023	0.450	0.246
	Two-tailed p-value	0.697	0.211	0.705	0.950	0.254	0.569	0.005	0.268		0.295	0.949	0.191	0.492
	N	10	10	10	10	10	10	10	10	10	10	10	10	10
Total power	Pearson correlation	.656*	0.286	-0.632	0.319	0.041	-0.307	.785**	0.219	0.368	1	0.319	0.255	0.327
	Two-tailed p-value	0.039	0.423	0.050	0.369	0.911	0.389	0.007	0.544	0.295		0.368	0.478	0.356
	N	10	10	10	10	10	10	10	10	10	10	10	10	10
SD1	Pearson correlation	.805**	0.572	-.794**	1.000**	.690*	0.000	0.422	.837**	0.023	0.319	1	0.363	0.164
	Two-tailed p-value	0.005	0.084	0.006	0.000	0.027	1.000	0.225	0.003	0.949	0.368		0.303	0.650
	N	10	10	10	10	10	10	10	10	10	10	10	10	10
SD2	Pearson correlation	0.502	.959**	-0.501	0.362	0.287	-0.349	0.525	0.129	0.450	0.255	0.363	1	0.104

	**Two-tailed p-value**	0.139	0.000	0.140	0.304	0.422	0.323	0.119	0.723	0.191	0.478	0.303		0.776
	**N**	10	10	10	10	10	10	10	10	10	10	10	10	10
**SD1toSD2ratio**	**Pearson correlation**	0.309	0.261	-0.292	0.164	0.004	-0.194	0.349	-0.156	0.246	0.327	0.164	0.104	1
	**Two-tailed p-value**	0.385	0.467	0.412	0.651	0.991	0.590	0.324	0.667	0.492	0.356	0.650	0.776	
	**N**	10	10	10	10	10	10	10	10	10	10	10	10	10

**. Correlation is significant at the 0.01 level (Two-tailed).

*. Correlation is significant at the 0.05 level (Two-tailed).

Table S5. DHVS-based HRV correlation matrix: Positive surprise

		Mean RR	SDNN	Mean HR	RMSSD	LF peak	HF peak	LF power	HF power	LFtoHF ratio	Total power	SD1	SD2	SD1to SD2 ratio
Mean RR	Pearson correlation	1	0.085	-0.299	-0.005	0.025	-0.012	0.436	-0.420	0.472	-0.108	0.024	0.095	-0.110
	Two-tailed p-value		0.816	0.402	0.989	0.945	0.975	0.208	0.226	0.169	0.767	0.947	0.794	0.762
	N	10	10	10	10	10	10	10	10	10	10	10	10	10
SDNN	Pearson correlation	0.085	1	0.382	.971**	0.100	-0.188	0.409	0.546	0.028	.746*	.943**	.998**	-0.213
	Two-tailed p-value	0.816		0.275	0.000	0.784	0.602	0.240	0.103	0.939	0.013	0.000	0.000	0.555
	N	10	10	10	10	10	10	10	10	10	10	10	10	10
Mean HR	Pearson correlation	-0.299	0.382	1	0.302	0.247	-0.369	0.213	0.066	0.300	0.083	0.448	0.358	0.234
	Two-tailed p-value	0.402	0.275		0.396	0.492	0.294	0.554	0.856	0.400	0.821	0.194	0.309	0.515
	N	10	10	10	10	10	10	10	10	10	10	10	10	10
RMSSD	Pearson correlation	-0.005	.971**	0.302	1	0.144	-0.108	0.326	.684*	-0.130	.826**	.943**	.963**	-0.148
	Two-tailed p-value	0.989	0.000	0.396		0.691	0.766	0.359	0.029	0.721	0.003	0.000	0.000	0.684
	N	10	10	10	10	10	10	10	10	10	10	10	10	10
LF peak	Pearson correlation	0.025	0.100	0.247	0.144	1	-0.072	0.446	0.017	0.422	0.088	0.210	0.068	0.291
	Two-tailed p-value	0.945	0.784	0.492	0.691		0.844	0.197	0.963	0.225	0.810	0.561	0.852	0.414
	N	10	10	10	10	10	10	10	10	10	10	10	10	10
HF peak	Pearson correlation	-0.012	-0.188	-0.369	-0.108	-0.072	1	-0.385	0.071	-0.433	-0.121	-0.148	-0.192	0.166

	**Two-tailed p-value**	0.975	0.602	0.294	0.766	0.844		0.272	0.846	0.211	0.740	0.684	0.595	0.646
	**N**	10	10	10	10	10	10	10	10	10	10	10	10	10
**LFpower**	**Pearson correlation**	0.436	0.409	0.213	0.326	0.446	-0.385	1	0.162	.817**	0.469	0.354	0.417	-0.211
	**Two-tailed p-value**	0.208	0.240	0.554	0.359	0.197	0.272		0.655	0.004	0.171	0.315	0.230	0.559
	**N**	10	10	10	10	10	10	10	10	10	10	10	10	10
**HF power**	**Pearson correlation**	-0.420	0.546	0.066	.684*	0.017	0.071	0.162	1	-0.373	.915**	0.533	0.545	-0.160
	**Two-tailed p-value**	0.226	0.103	0.856	0.029	0.963	0.846	0.655		0.289	0.000	0.113	0.104	0.660
	**N**	10	10	10	10	10	10	10	10	10	10	10	10	10
**LFtoHFratio**	**Pearson correlation**	0.472	0.028	0.300	-0.130	0.422	-0.433	.817**	-0.373	1	-0.090	-0.034	0.039	-0.152
	**Two-tailed p-value**	0.169	0.939	0.400	0.721	0.225	0.211	0.004	0.289		0.804	0.927	0.914	0.674
	**N**	10	10	10	10	10	10	10	10	10	10	10	10	10
**Total power**	**Pearson correlation**	-0.108	.746*	0.083	.826**	0.088	-0.121	0.469	.915**	-0.090	1	.694*	.750*	-0.273
	**Two-tailed p-value**	0.767	0.013	0.821	0.003	0.810	0.740	0.171	0.000	0.804		0.026	0.012	0.446
	**N**	10	10	10	10	10	10	10	10	10	10	10	10	10
**SD1**	**Pearson correlation**	0.024	.943**	0.448	.943**	0.210	-0.148	0.354	0.533	-0.034	.694*	1	.919**	0.120
	**Two-tailed p-value**	0.947	0.000	0.194	0.000	0.561	0.684	0.315	0.113	0.927	0.026		0.000	0.742
	**N**	10	10	10	10	10	10	10	10	10	10	10	10	10
**SD2**	**Pearson correlation**	0.095	.998**	0.358	.963**	0.068	-0.192	0.417	0.545	0.039	.750*	.919**	1	-0.276

	**Two-tailed p-value**	0.794	0.000	0.309	0.000	0.852	0.595	0.230	0.104	0.914	0.012	0.000		0.441
	**N**	10	10	10	10	10	10	10	10	10	10	10	10	10
**SD1toSD2ratio**	**Pearson correlation**	-0.110	-0.213	0.234	-0.148	0.291	0.166	-0.211	-0.160	-0.152	-0.273	0.120	-0.276	1
	**Two-tailed p-value**	0.762	0.555	0.515	0.684	0.414	0.646	0.559	0.660	0.674	0.446	0.742	0.441	
	**N**	10	10	10	10	10	10	10	10	10	10	10	10	10

**. Correlation is significant at the 0.01 level (Two-tailed).

*. Correlation is significant at the 0.05 level (Two-tailed).

Table S6. DHVS-based HRV correlation matrix: Positive sadness

		Mean RR	SDNN	Mean HR	RMSSD	LF peak	HF peak	LF power	HF power	LFtoHF ratio	Total power	SD1	SD2	SD1to SD2 ratio
**Mean RR**	**Pearson correlation**	1	0.292	-0.450	0.224	0.313	-0.120	0.124	0.023	-0.138	0.292	0.297	0.203	0.362
	**Two-tailed p-value**		0.484	0.263	0.594	0.451	0.777	0.769	0.957	0.744	0.482	0.475	0.630	0.378
	**N**	8	8	8	8	8	8	8	8	8	8	8	8	8
**SDNN**	**Pearson correlation**	0.292	1	-0.385	.993**	-0.208	-0.679	0.650	0.417	-0.111	.967**	.982**	.971**	.862**
	**Two-tailed p-value**	0.484		0.347	0.000	0.622	0.064	0.081	0.304	0.793	0.000	0.000	0.000	0.006
	**N**	8	8	8	8	8	8	8	8	8	8	8	8	8
**Mean HR**	**Pearson correlation**	-0.450	-0.385	1	-0.310	0.189	0.538	-0.678	-0.596	-0.274	-0.534	-0.398	-0.434	-0.360
	**Two-tailed p-value**	0.263	0.347		0.456	0.653	0.169	0.065	0.119	0.511	0.173	0.329	0.283	0.381
	**N**	8	8	8	8	8	8	8	8	8	8	8	8	8
**RMSSD**	**Pearson correlation**	0.224	.993**	-0.310	1	-0.159	-0.632	0.612	0.384	-0.132	.948**	.980**	.954**	.874**
	**Two-tailed p-value**	0.594	0.000	0.456		0.706	0.093	0.107	0.348	0.755	0.000	0.000	0.000	0.005
	**N**	8	8	8	8	8	8	8	8	8	8	8	8	8
**LF peak**	**Pearson correlation**	0.313	-0.208	0.189	-0.159	1	0.548	-0.373	-0.413	-0.157	-0.243	-0.177	-0.415	0.022
	**Two-tailed p-value**	0.451	0.622	0.653	0.706		0.159	0.363	0.309	0.711	0.562	0.674	0.306	0.958
	**N**	8	8	8	8	8	8	8	8	8	8	8	8	8
**HF peak**	**Pearson correlation**	-0.120	-0.679	0.538	-0.632	0.548	1	-.946**	-0.160	-0.572	-0.671	-0.625	-.723*	-0.433

	**Two-tailed p-value**	0.777	0.064	0.169	0.093	0.159		0.000	0.705	0.139	0.069	0.097	0.043	0.284
	**N**	8	8	8	8	8	8	8	8	8	8	8	8	8
**LFpower**	**Pearson correlation**	0.124	0.650	-0.678	0.612	-0.373	-.946**	1	0.220	0.626	0.686	0.604	0.665	0.448
	**Two-tailed p-value**	0.769	0.081	0.065	0.107	0.363	0.000		0.601	0.097	0.060	0.112	0.072	0.265
	**N**	8	8	8	8	8	8	8	8	8	8	8	8	8
**HF power**	**Pearson correlation**	0.023	0.417	-0.596	0.384	-0.413	-0.160	0.220	1	-0.424	0.580	0.441	0.542	0.362
	**Two-tailed p-value**	0.957	0.304	0.119	0.348	0.309	0.705	0.601		0.295	0.132	0.274	0.165	0.378
	**N**	8	8	8	8	8	8	8	8	8	8	8	8	8
**LFtoHFratio**	**Pearson correlation**	-0.138	-0.111	-0.274	-0.132	-0.157	-0.572	0.626	-0.424	1	-0.126	-0.141	-0.109	-0.187
	**Two-tailed p-value**	0.744	0.793	0.511	0.755	0.711	0.139	0.097	0.295		0.766	0.739	0.797	0.658
	**N**	8	8	8	8	8	8	8	8	8	8	8	8	8
**Total power**	**Pearson correlation**	0.292	.967**	-0.534	.948**	-0.243	-0.671	0.686	0.580	-0.126	1	.934**	.955**	.784*
	**Two-tailed p-value**	0.482	0.000	0.173	0.000	0.562	0.069	0.060	0.132	0.766		0.001	0.000	0.021
	**N**	8	8	8	8	8	8	8	8	8	8	8	8	8
**SD1**	**Pearson correlation**	0.297	.982**	-0.398	.980**	-0.177	-0.625	0.604	0.441	-0.141	.934**	1	.961**	.937**
	**Two-tailed p-value**	0.475	0.000	0.329	0.000	0.674	0.097	0.112	0.274	0.739	0.001		0.000	0.001
	**N**	8	8	8	8	8	8	8	8	8	8	8	8	8
**SD2**	**Pearson correlation**	0.203	.971**	-0.434	.954**	-0.415	-.723*	0.665	0.542	-0.109	.955**	.961**	1	.822*

	**Two-tailed p-value**	0.630	0.000	0.283	0.000	0.306	0.043	0.072	0.165	0.797	0.000	0.000		0.012
	**N**	8	8	8	8	8	8	8	8	8	8	8	8	8
**SD1toSD2ratio**	**Pearson correlation**	0.362	.862**	-0.360	.874**	0.022	-0.433	0.448	0.362	-0.187	.784*	.937**	.822*	1
	**Two-tailed p-value**	0.378	0.006	0.381	0.005	0.958	0.284	0.265	0.378	0.658	0.021	0.001	0.012	
	**N**	8	8	8	8	8	8	8	8	8	8	8	8	8

**. Correlation is significant at the 0.01 level (Two-tailed).

*. Correlation is significant at the 0.05 level (Two-tailed).

Table S7. DHVS-based HRV correlation matrix: Negative surprise

		Mean RR	SDNN	Mean HR	RMSSD	LF peak	HF peak	LF power	HF power	LFtoHF ratio	Total power	SD1	SD2	SD1to SD2 ratio
Mean RR	Pearson correlation	1	0.087	-0.314	-0.082	0.018	-0.429	0.242	0.203	0.063	0.254	0.052	0.139	0.032
	Two-tailed p-value		0.824	0.411	0.835	0.963	0.249	0.531	0.600	0.872	0.510	0.895	0.721	0.935
	N	9	9	9	9	9	9	9	9	9	9	9	9	9
SDNN	Pearson correlation	0.087	1	-0.183	.776*	0.257	-0.392	0.433	.785*	-0.307	.689*	0.510	.969**	-0.097
	Two-tailed p-value	0.824		0.638	0.014	0.504	0.297	0.244	0.012	0.422	0.040	0.160	0.000	0.805
	N	9	9	9	9	9	9	9	9	9	9	9	9	9
Mean HR	Pearson correlation	-0.314	-0.183	1	-0.083	-0.653	.790*	-0.262	-0.303	-0.172	-0.281	-0.146	-0.173	-0.055
	Two-tailed p-value	0.411	0.638		0.831	0.056	0.011	0.496	0.429	0.657	0.465	0.707	0.657	0.888
	N	9	9	9	9	9	9	9	9	9	9	9	9	9
RMSSD	Pearson correlation	-0.082	.776*	-0.083	1	-0.121	-0.049	-0.150	0.319	-0.495	0.128	.893**	0.607	0.485
	Two-tailed p-value	0.835	0.014	0.831		0.757	0.899	0.700	0.402	0.176	0.743	0.001	0.083	0.185
	N	9	9	9	9	9	9	9	9	9	9	9	9	9
LF peak	Pearson correlation	0.018	0.257	-0.653	-0.121	1	-.871**	0.548	0.537	0.401	0.537	-0.268	0.338	-0.500
	Two-tailed p-value	0.963	0.504	0.056	0.757		0.002	0.127	0.136	0.285	0.136	0.486	0.373	0.171
	N	9	9	9	9	9	9	9	9	9	9	9	9	9
HF peak	Pearson correlation	-0.429	-0.392	.790*	-0.049	-.871**	1	-0.594	-0.590	-0.401	-0.605	0.051	-0.461	0.327

	**Two-tailed p-value**	0.249	0.297	0.011	0.899	0.002		0.091	0.094	0.285	0.084	0.896	0.212	0.390
	**N**	9	9	9	9	9	9	9	9	9	9	9	9	9
**LFpower**	**Pearson correlation**	0.242	0.433	-0.262	-0.150	0.548	-0.594	1	.750*	0.488	.928**	-0.445	0.628	-.774*
	**Two-tailed p-value**	0.531	0.244	0.496	0.700	0.127	0.091		0.020	0.182	0.000	0.230	0.070	0.014
	**N**	9	9	9	9	9	9	9	9	9	9	9	9	9
**HF power**	**Pearson correlation**	0.203	.785*	-0.303	0.319	0.537	-0.590	.750*	1	-0.098	.935**	-0.022	.870**	-0.561
	**Two-tailed p-value**	0.600	0.012	0.429	0.402	0.136	0.094	0.020		0.801	0.000	0.956	0.002	0.116
	**N**	9	9	9	9	9	9	9	9	9	9	9	9	9
**LFtoHFratio**	**Pearson correlation**	0.063	-0.307	-0.172	-0.495	0.401	-0.401	0.488	-0.098	1	0.166	-0.548	-0.159	-0.399
	**Two-tailed p-value**	0.872	0.422	0.657	0.176	0.285	0.285	0.182	0.801		0.670	0.127	0.683	0.287
	**N**	9	9	9	9	9	9	9	9	9	9	9	9	9
**Total power**	**Pearson correlation**	0.254	.689*	-0.281	0.128	0.537	-0.605	.928**	.935**	0.166	1	-0.204	.834**	-.685*
	**Two-tailed p-value**	0.510	0.040	0.465	0.743	0.136	0.084	0.000	0.000	0.670		0.598	0.005	0.042
	**N**	9	9	9	9	9	9	9	9	9	9	9	9	9
**SD1**	**Pearson correlation**	0.052	0.510	-0.146	.893**	-0.268	0.051	-0.445	-0.022	-0.548	-0.204	1	0.293	.803**
	**Two-tailed p-value**	0.895	0.160	0.707	0.001	0.486	0.896	0.230	0.956	0.127	0.598		0.444	0.009
	**N**	9	9	9	9	9	9	9	9	9	9	9	9	9
**SD2**	**Pearson correlation**	0.139	.969**	-0.173	0.607	0.338	-0.461	0.628	.870**	-0.159	.834**	0.293	1	-0.320

	**Two-tailed p-value**	0.721	0.000	0.657	0.083	0.373	0.212	0.070	0.002	0.683	0.005	0.444		0.401
	**N**	9	9	9	9	9	9	9	9	9	9	9	9	9
**SD1toSD2ratio**	**Pearson correlation**	0.032	-0.097	-0.055	0.485	-0.500	0.327	-.774*	-0.561	-0.399	-.685*	.803**	-0.320	1
	**Two-tailed p-value**	0.935	0.805	0.888	0.185	0.171	0.390	0.014	0.116	0.287	0.042	0.009	0.401	
	**N**	9	9	9	9	9	9	9	9	9	9	9	9	9

**. Correlation is significant at the 0.01 level (Two-tailed).

*. Correlation is significant at the 0.05 level (Two-tailed).

Table S8. DHVS-based HRV correlation matrix: Negative sadness

		Mean RR	SDNN	Mean HR	RMSSD	LF peak	HF peak	LF power	HF power	LFtoHF ratio	Total power	SD1	SD2	SD1to SD2 ratio
Mean RR	Pearson correlation	1	0.420	0.011	0.407	-0.100	0.247	0.311	-0.059	0.620	0.018	0.298	0.430	-0.033
	Two-tailed p-value		0.227	0.976	0.244	0.784	0.492	0.381	0.870	0.056	0.961	0.403	0.215	0.928
	N	10	10	10	10	10	10	10	10	10	10	10	10	10
SDNN	Pearson correlation	0.420	1	-0.467	.978**	-0.166	0.100	0.565	.716*	0.064	.766**	.865**	.994**	0.010
	Two-tailed p-value	0.227		0.173	0.000	0.647	0.783	0.089	0.020	0.860	0.010	0.001	0.000	0.977
	N	10	10	10	10	10	10	10	10	10	10	10	10	10
Mean HR	Pearson correlation	0.011	-0.467	1	-0.440	0.345	-0.156	-0.379	-0.143	-0.194	-0.212	-0.236	-0.506	0.353
	Two-tailed p-value	0.976	0.173		0.203	0.329	0.667	0.279	0.694	0.591	0.557	0.512	0.135	0.317
	N	10	10	10	10	10	10	10	10	10	10	10	10	10
RMSSD	Pearson correlation	0.407	.978**	-0.440	1	-0.246	0.231	0.469	.680*	0.003	.707*	.849**	.970**	0.031
	Two-tailed p-value	0.244	0.000	0.203		0.493	0.522	0.171	0.031	0.993	0.022	0.002	0.000	0.932
	N	10	10	10	10	10	10	10	10	10	10	10	10	10
LF peak	Pearson correlation	-0.100	-0.166	0.345	-0.246	1	-0.337	0.499	0.183	0.263	0.353	-0.238	-0.163	-0.194
	Two-tailed p-value	0.784	0.647	0.329	0.493		0.341	0.142	0.613	0.464	0.317	0.509	0.652	0.592
	N	10	10	10	10	10	10	10	10	10	10	10	10	10
HF peak	Pearson correlation	0.247	0.100	-0.156	0.231	-0.337	1	-0.044	-0.376	0.172	-0.326	0.000	0.119	-0.028

	Two-tailed p-value	0.492	0.783	0.667	0.522	0.341		0.903	0.284	0.634	0.358	0.999	0.744	0.939
	N	10	10	10	10	10	10	10	10	10	10	10	10	10
LFpower	Pearson correlation	0.311	0.565	-0.379	0.469	0.499	-0.044	1	0.368	0.594	0.616	0.332	0.581	-0.272
	Two-tailed p-value	0.381	0.089	0.279	0.171	0.142	0.903		0.295	0.070	0.058	0.349	0.078	0.447
	N	10	10	10	10	10	10	10	10	10	10	10	10	10
HF power	Pearson correlation	-0.059	.716*	-0.143	.680*	0.183	-0.376	0.368	1	-0.362	.952**	.755*	.676*	0.169
	Two-tailed p-value	0.870	0.020	0.694	0.031	0.613	0.284	0.295		0.303	0.000	0.012	0.032	0.640
	N	10	10	10	10	10	10	10	10	10	10	10	10	10
LFtoHFratio	Pearson correlation	0.620	0.064	-0.194	0.003	0.263	0.172	0.594	-0.362	1	-0.144	-0.280	0.125	-0.592
	Two-tailed p-value	0.056	0.860	0.591	0.993	0.464	0.634	0.070	0.303		0.691	0.433	0.730	0.071
	N	10	10	10	10	10	10	10	10	10	10	10	10	10
Total power	Pearson correlation	0.018	.766**	-0.212	.707*	0.353	-0.326	0.616	.952**	-0.144	1	.718*	.740*	0.047
	Two-tailed p-value	0.961	0.010	0.557	0.022	0.317	0.358	0.058	0.000	0.691		0.019	0.014	0.898
	N	10	10	10	10	10	10	10	10	10	10	10	10	10
SD1	Pearson correlation	0.298	.865**	-0.236	.849**	-0.238	0.000	0.332	.755*	-0.280	.718*	1	.811**	0.498
	Two-tailed p-value	0.403	0.001	0.512	0.002	0.509	0.999	0.349	0.012	0.433	0.019		0.004	0.143
	N	10	10	10	10	10	10	10	10	10	10	10	10	10
SD2	Pearson correlation	0.430	.994**	-0.506	.970**	-0.163	0.119	0.581	.676*	0.125	.740*	.811**	1	-0.086

	**Two-tailed p-value**	0.215	0.000	0.135	0.000	0.652	0.744	0.078	0.032	0.730	0.014	0.004		0.814
	**N**	10	10	10	10	10	10	10	10	10	10	10	10	10
**SD1toSD2ratio**	**Pearson correlation**	-0.033	0.010	0.353	0.031	-0.194	-0.028	-0.272	0.169	-0.592	0.047	0.498	-0.086	1
	**Two-tailed p-value**	0.928	0.977	0.317	0.932	0.592	0.939	0.447	0.640	0.071	0.898	0.143	0.814	
	**N**	10	10	10	10	10	10	10	10	10	10	10	10	10

**. Correlation is significant at the 0.01 level (Two-tailed). *. Correlation is significant at the 0.05 level (Two-tailed).
